# A cross-sectional study exploring the perception of exercise oncology in the Italian population

**DOI:** 10.3389/fonc.2024.1430083

**Published:** 2025-01-13

**Authors:** Anita Borsati, Diana Giannarelli, Giampaolo Pase, Christian Ciurnelli, Linda Toniolo, Ilaria Trestini, Daniela Tregnago, Lorenzo Belluomini, Marco Sposito, Jessica Insolda, Michele Milella, Federico Schena, Sara Pilotto, Alice Avancini

**Affiliations:** ^1^ Department of Medicine, University of Verona, Verona, Italy; ^2^ Fondazione Policlinico Universitario A. Gemelli, Istituto di Ricovero e Cura a Carattere Scientifico (IRCCS)-Epidemiology and Biostatistic, Rome, Italy; ^3^ Department of Neurosciences, Biomedicine and Movement, University of Verona, Verona, Italy; ^4^ Dietetic Service, Medical Direction, University Hospital of Verona (AOUI), Verona, Italy; ^5^ Section of Innovation Biomedicine - Oncology Area, Department of Engineering for Innovation Medicine (DIMI), University of Verona and University and Hospital Trust (AOUI) of Verona, Verona, Italy

**Keywords:** physical exercise, cancer, population perception, stigma, patients with cancer

## Abstract

**Introduction:**

We aim to examine the population’s perception of physical exercise in patients with cancer.

**Materials and methods:**

An anonymous survey was conducted to reach a sample of Italian adults. The questionnaire investigated sociodemographic factors, physical exercise levels, and perceptions about the importance, benefits, and safety of exercise, the support from oncologists and family/friends, as well as the capability and ease of patients of exercise.

**Results:**

Overall, 838 persons participated in this survey. The majority of respondents agree that exercise is important (60.5%) and beneficial (61.5%) for patients with cancer during anticancer treatments, whereas 40.2% believed in its safety. Forty-two percent and 51.9% of participants expressed a positive opinion regarding the advice of oncologists and the encouragement of family/friends to exercise, respectively. Only 27.2% of respondents feel that patients are capable of exercising, and 9.0% agree that it is easy for them.

**Conclusion:**

Although the population has a favorable perception of the importance and benefits of physical exercise, they do not still believe that patients are capable of performing it. Increasing awareness of the feasibility of a physical exercise intervention in the context of cancer is crucial to supporting patients.

## Introduction

Even just the word *cancer* inspires fear among the population. Despite the several advances in terms of early diagnosis, treatments benefit and tolerability, and quality of life, more than half of the general population is afraid of cancer more than of other diseases (1). Cancer is associated with negative emotions and thoughts, such as death, suffering, pain, helplessness, long and sickening treatments, and unpredictability (2). For the population, the typical profile of a patient with cancer can be summarized as a bald person with tiredness and muscle weakness who needs continuous help due to their progressive loss of independence (3). These cultural beliefs have led to the stigmatization of patients with cancer, isolating them from social life and reinforcing the misconception that they cannot engage in physical activity (4). Indeed, for a long time, physical activity (i.e., any voluntary bodily movement produced by skeletal muscles that requires energy expenditure) and physical exercise (i.e., planned, structured, and repetitive physical activity aiming to improve physical fitness) have been discouraged in patients with cancer (5, 6).

Nevertheless, over the past 30 years, the research on *exercise-oncology* has exponentially increased, supporting the beneficial impact of physical activity and exercise on several cancer-related aspects. Epidemiological evidence reveals an inverse association between physical activity performed after diagnosis and mortality, with reductions greater than 40% for all-cause and cancer-specific mortality (7). Recently, this correlation has also been found in patients undergoing innovative treatments, such as immunotherapy (8). Beyond the impact on survival, interventional studies on physical exercise demonstrated its optimal safety profile and its beneficial effect in improving physical fitness components (i.e., cardiorespiratory fitness (9, 10), strength (11), body composition (11), flexibility), managing treatment-related side effects (8, 12), and enhancing psychological outcomes such as anxiety and depression (13). Overall, engaging in physical exercise after a diagnosis significantly ameliorates patients’ quality of life from physical, emotional, and social points of view (12, 14).

Different scientific societies strongly recommend that patients with cancer should engage in regular physical activity (5, 15, 16). The American College of Sports Medicine (ACSM) advises patients to perform 90 minutes per week of moderate-intensity aerobic activity, adding strength training twice a week (5). Based on this guideline, a survey investigated physical exercise levels in 324 patients with cancer found that only 4% of them met the current recommendations (17).

As suggested by behavioral science theory, no single factor accounts for why patients do not engage in sufficient physical exercise (18). Embracing an ecological perspective, several personal, interpersonal, environmental, and policy aspects may contribute to the development, maintenance, and change of physical exercise patterns (18). On a personal level, attitude, knowledge, and motivation are key determinants of physical exercise. In this light, different researchers have investigated the just-mentioned issue in patients with cancer (19–21). Additionally, as postulated by the social-ecological model, behavior, such as physical exercise, shapes and is shaped by the social environment (18). Therefore, translating these concepts into an exercise-oncology setting, the social relations, including caregivers’ and healthcare providers’ support, and the cultural environment, such as the perceptions of the community about physical exercise for patients with cancer, may play a significant role in influencing physical exercise among patients (22–26). For instance, the involvement of family, friends, and community can significantly impact patients’ willingness to engage in physical exercise during and after cancer treatment (23). On the cultural front, community beliefs about illness and treatment may discourage the adoption of healthy behavior (27). In the past, patients with cancer were advised to rest and avoid physical activity due to concerns about worsening their condition, a view rooted in traditional beliefs that saw cancer as a condition requiring rest. This credence might still be present and negatively influence the engagement of patients in physical exercise.

In the literature, there is already available information about attitudes and perceptions regarding exercise oncology among caregivers and healthcare providers (28–30), but to our knowledge, no studies so far have focused on the general population. Therefore, recognizing the potential impact of cultural environment and beliefs on behavior, (i.e., physical exercise in patients with cancer), and given the lack of data on this topic, we designed the Population Perception Exercise Oncology (POPCORN) study. The primary aim of the study was to explore the perception of the Italian population about physical exercise in patients with cancer undergoing anticancer treatments. The secondary aim was to identify the respondents’ characteristics associated with a positive/neutral/negative perception of exercise oncology.

## Materials and methods

### Study design, participants, and procedures

The POPCORN study was a cross-sectional anonymous survey delivered via Facebook between September 2023 and October 2023. Participants’ eligibility criteria were age ≥ 18 years old, having a Facebook account, being Italian-speaking, and residing in Italy. The decision to spread the survey only using Facebook was based on several reasons: first, Facebook is the most widely used social network in Italy and globally, with a balanced distribution across age groups in the population (31). Additionally, studies comparing various social media platforms in the United States have indicated that Facebook is the most representative in terms of users’ educational attainment and internet skills (32). Finally, Facebook offers the significant advantage of enabling targeted advertisements, allowing us to reach specific audiences efficiently while being both time- and cost-effective (33, 34). Participants were recruited online through a Facebook advertisement. A tailored advertisement was utilized to reach Facebook users who matched the eligibility criteria and try to obtain a more representative sample of the Italian population. When participants clicked on the Facebook advertisement, they were directed to a secure Google^®^ form, providing the study description and informed consent. Informed consent was provided by participants before starting the survey; after signing the informed consent, participants were redirected to the pre-screening section and asked to provide their age and residence. If eligible, participants were addressed to the questionnaire. Data were securely kept on a password-protected computer. The Facebook Advertisement Manager was utilized to track the total number of impressions (i.e., the number of times that advertisement was displayed) and click on the advertisement. No incentives were offered for participation.

The present study adhered to Good Clinical Practice principles. All the procedures were conducted in compliance with the Helsinki and Oviedo declarations. The Verona University Ethics Committee has reviewed and approved the study (Prot. N. 02/2023). To ensure transparency in the study design and in the recruitment process, we followed the Strengthening the Reporting of Observational Studies in Epidemiology (STROBE) (35) and the Checklist for Reporting Results of Internet E-Surveys (CHERRIES) guidelines (36) to report the study results.

### Questionnaire

The POPCORN questionnaire was designed to assess the Italian population’s perception toward exercise for patients with cancer undergoing anticancer treatments. The survey was developed using a co-design process involving patients and experts such as kinesiologists, oncologists, and psychologists and based on the current literature (29, 37). The questionnaire was composed of 24 items and divided into three sections: i) general characteristics, ii) physical activity level, iii) perception about physical exercise in patients with cancer undergoing anticancer treatments.

The participants’ general characteristics included sex (male/female), birth date (month/day/year), occupational status (retired/homemaker/part-time employed/full-time employed), perceived economic adequacy (inadequate/barely adequate/adequate/more than adequate), current or past cancer diagnosis (yes/no), and being a healthcare provider working on cancer patients (yes/no).

The Godin Leisure Time Exercise Questionnaire was used to explore the physical activity level of participants. The questionnaire was composed of three questions asking the frequency of vigorous, moderate, and mild-intensity activities for at least 15 minutes in a typical week. Each intensity is related to a metabolic equivalent of the task (MET), 9 for vigorous, 5 for moderate, and 3 for mild intensity exercise. According to Godin and Shepard, the Leisure Score Index (LSI) was calculated as follows (frequency of vigorous * 9) + (frequency of moderate * 5) to identify physically active and inactive people. An LSI ≥ 24 indicates an active individual, whereas an individual with an LSI < 24 was considered insufficiently active according to the current physical activity guidelines. The questionnaire comprised an additional closed question about the frequency (times/week) of sweat-inducing activity (often/sometimes/never–rarely) (38).

Perception of physical exercise in patients with cancer undergoing treatments was explored using questions drawn and adapted to the context of a general population, from a prior study and based on the Theory of Planned Behavior (37). This behavioral model sustains the hypothesis that the intention of an individual to perform a behavior is influenced and determined by three independent constructs: the attitude (i.e., the positive or negative evaluation of performing the behavior), subjective norm (i.e., the perceived social pressure that individuals may feel to perform or not a behavior), and perceived behavioral control (i.e., perception of ease or difficulty of performing the behavior). Attitude was investigated through three items by asking participants if they perceive physical exercise for patients with cancer as beneficial, important, and safe. Whether oncologists and family or friends should encourage patients to exercise during treatments was used to assess the subjective norm (two items), whereas perceived behavioral control was investigated by asking if, in their opinion, patients are capable of exercising and it is easy for them to engage in regular exercise (two items). A 7-point Likert scale from 1 (strongly disagree) to 7 (strongly agree) was used to assess all items. The scale displayed a high internal consistency (Cronbach’s alpha, α = 0.86). To present data based on prior literature, the items were grouped into three categories: disagree (1-2), neutral (3-5), and agree (6-7) (37). As a final question, participants were asked, in their opinion, what is the percentage of patients that regularly exercise during anticancer treatments, through one close question (<20%/20-40%/40-60%/60-80%/>80%).

### Analysis

Participants’ characteristics are reported as absolute counts and percentages. The association between the questionnaire responses and participants’ features is assessed by the chi-square test. A multivariable generalized linear model was implemented to investigate the correlation between each scale and respondents’ traits. When positive, beta coefficients suggest an increase in agreement, while negative values indicate an increase in disagreement; significance levels are calculated using the Wald test. Analyses were performed using IBM SPSS Statistics, v.28.0.

## Results

The social media advertisement was visualized by 23.667 accounts. Of the 943 individuals who clicked on the link redirecting them to the survey, 838 met the eligibility criteria and completed the questionnaire.

**Table 1** encloses the sociodemographic characteristics of the participants. In brief, more than half of the respondents (65.2%) were female, median age was 39 years old, 84.9% lived in the northern regions of Italy, and 58.4% had a full-time employment occupation. About 15.8% of participants declared to have or have had a diagnosis of cancer, 13.4% were healthcare providers working within the cancer context, and 28.5% were sufficiently active, according to LSI.

**Table 1:** Socio-demographic characteristics of the study participants.

Variable	Number	Percentage
Gender		
Female	546	65.2
Male	292	34.8
Age		
< 39	408	48.7
≥ 39	429	52.3
Region		
North	697	84.9
Central-South	124	15.1
Occupational status		
Full-time employed	489	58.4
Part-time employed	108	12.9
Seeking employment.	36	4.3
Retired	83	9.9
Homemaker	15	1.8
Others	106	12.7
Perceived income adequacy		
More than adequate	166	19.9
Adequate	406	48.6
Barely adequate	232	27.8
Inadequate	31	3.7
Current or past diagnosis of cancer		
Yes	132	15.8
No	705	84.2
Healthcare professional		
Yes	111	13.4
No	715	86.6
Exercise level		
Insufficiently active	599	72.5
Sufficiently active	239	28.5

### Population perception about physical exercise in patients with cancer during anticancer treatments

**Figure 1:**
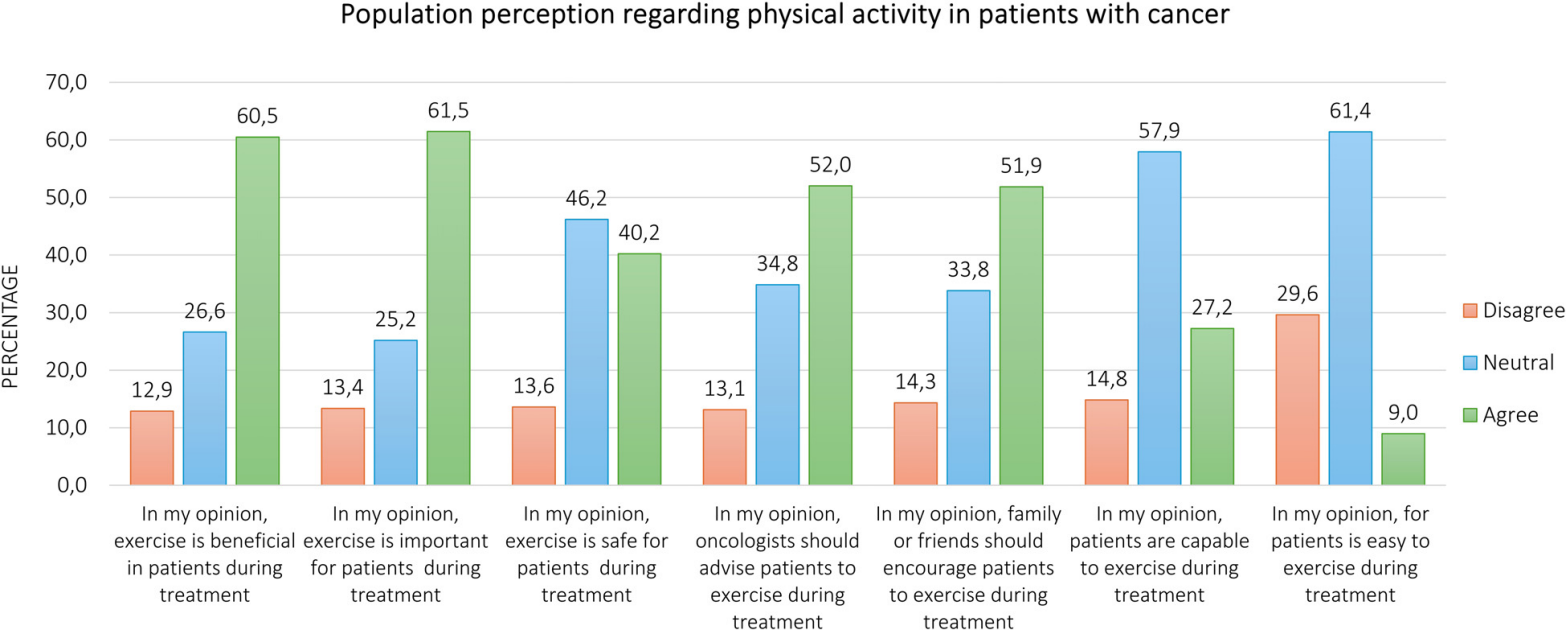
Population perception about physical exercise in patients with cancer during anticancer treatments.

**Figure 2:**
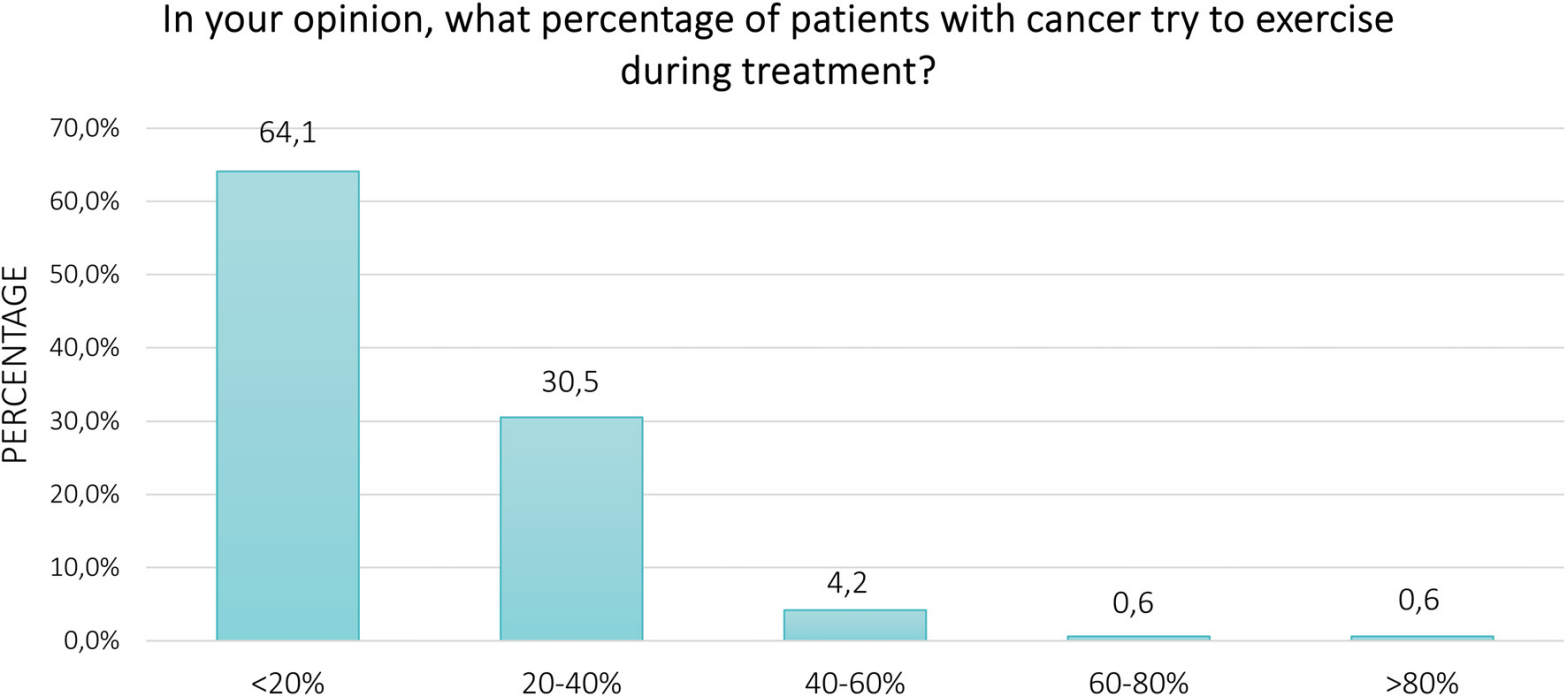
Percentage of patients with cancer believed to exercise during treatments.

Regarding the perception of the respondents about physical exercise in patients with cancer undergoing anticancer treatments, **Figure 1**. represents the proportion of agree, neutral, and disagree. Overall, the majority of participants agreed that physical exercise is beneficial (60.5%) and important (61.5%), whereas 40.2% perceived it to be safe for patients. About 52.0% of respondents thought that oncologists should advise patients to exercise, and 51.8% thought that family or friends should encourage them to engage in regular physical exercise. Conversely, only 27.2% and 8.9% of survey participants believed that patients are capable and it is easy for them to perform exercise, respectively. Fifty-eight percent and 61.5% of respondents provided neutral answers for these two items. Finally, 64.1% of participants felt that less than 20% of patients engage in physical exercise during anticancer treatment (**Figure 2**). Excluding the responders who were healthcare providers, the results remained aligned, showing no significant differences (**Supplementary Tables S8**, **S9**).

### Differences in physical exercise perception by sociodemographic characteristics and physical activity level

Numerous differences in perceptions emerged based on respondents’ sociographic characteristics (**Supplementary Tables S1**–**S7**). Male participants were more likely to express neutral opinions about the beneficial effects of exercise (32.4% vs. 23.1%, p = 0.007) and its importance in cancer (31.5% vs. 21.5%, p = 0.003). Similarly, those living in northern Italy (28.1% vs. 16.5%, p = 0.027) or without a cancer diagnosis (28.3% vs. 15.4%, p < 0.001) showed more neutral views. Non-healthcare providers also expressed higher neutrality regarding the benefits (27.6% vs. 15.5%, p = 0.016) and safety (48.3% vs. 31.5%, p = 0.004) of exercise. Respondents with a history of cancer were more likely to disagree with the safety of exercise (24.2% vs. 11.1%, p < 0.001) and with oncologists’ advice (22.7% vs. 11.4%, p = 0.001) or encouragement from family and friends (22.9% vs. 12.6%, p = 0.002). Conversely, sufficiently active participants reported higher agreement with oncologists’ advice (59.1% vs. 49.2%, p = 0.037) and family encouragement (60.5% vs. 48.6%, p = 0.008). Healthcare providers similarly expressed more agreement regarding the role of oncologists in promoting exercise (60.4% vs. 50.8%, p = 0.003). Geographical differences were also evident, with participants from Central and Southern Italy showing more agreement about patients’ capability to exercise (37.4% vs. 25.5%, p = 0.018), while those in northern Italy (60.0% vs. 48.0%, p = 0.018) and those without financial difficulties (61.8% vs. 49.8%, p = 0.001) expressed more neutral opinions. The **Supplementary Materials** (**Supplementary Tables S11**–**S17**) reported the findings, excluding the healthcare providers’ samples; no significant differences in perceptions of oncologists’ recommendations or family encouragement based on gender, region, financial status, or physical activity levels emerged.

### Relationship between socio-economic variables within population perception

**Table 2** shows how respondents’ characteristics were related to their perceptions about physical exercise in cancer. The agreement regarding the perception that it is easy for patients to practice physical exercise was higher in respondents aged >39 years (Β=0.30, SE=0.12, p=0.01) than in those aged ≤ 39. Compared with participants in northern Italy, those who lived in Central South have higher agreement about the capability of patients to exercise (Β=0.37, SE=0.16, p=0.02), and the thought that it is easy for them to perform exercise (Β=0.46, SE=0.12, p=0.01). Compared to respondents who perceived their financial income as inadequate, those who reported it as adequate showed more agreement about the perceptions of exercise as beneficial (Β=0.36, SE=0.14, p=0.01), important (Β=0.29, SE=0.14, p=0.04) and regarding the oncologist advice (Β=0.34, SE=0.14, p=0.01). Finally, agreement regarding the perception of the capability of patients to exercise was higher in sufficiently active respondents (Β=0.28, SE=0.13, p=0.03) than in those who were insufficiently active. 

**Table 2:** Multivariable regression of associations of participants’ characteristics with their perception regarding exercise in cancer.

	Exercise is beneficial		Exercise is important		Exercise is safe		Patients are able to exercise		Oncologists should advise patients to exercise		Family/friends should advise patients to exercise		Exercising is easy for patients	
	Β (SE)	p-value	Β (SE)	p-value	Β (SE)	p-value	Β (SE)	p-value	Β (SE)	p-value	Β (SE)	p-value	Β (SE)	p-value
Gender														
Female	Ref		Ref		Ref		Ref		Ref		Ref		Ref	
Male	-0.09 (0.14)	0.52	-0.08 (0.14)	0.56	-0.13 (0.13)	0.33	-0.11 (0.13)	0.40	-0.10 (0.14)	0.46	-0.07 (0.14)	0.62	-0.01 (0.12)	0.92
Age														
≤ 39	Ref		Ref		Ref		Ref		Ref		Ref		Ref	
> 39	-0.22 (0.14)	0.11	-0.21 (0.14)	0.14	-0.02 (0.13)	0.90	0.08 (0.13)	0.53	-0.07 (0.14)	0.59	-0.24 (0.14)	0.09	0.30 (0.12)	0.01
Region														
North	Ref		Ref		Ref		Ref		Ref		Ref		Ref	
Central-South	0.22 (0.18)	0.23	0.18 (0.18)	0.33	0.18 (0.17)	0.30	0.37 (0.16)	0.02	0.21 (0.18)	0.23	-0.01 (0.18)	0.97	0.46 (0.15)	0.01
Occupation														
No	Ref		Ref		Ref		Ref		Ref		Ref		Ref	
Yes	0.02 (0.14)	0.91	0.01 (0.14)	0.93	0.06 (0.136)	0.68	-0.07 (0.13)	0.61	0.03 (0.14)	0.84	0.07 (0.15)	0.63	-0.01 (0.12)	0.97
Perceived income adequacy														
Inadequate	Ref		Ref		Ref		Ref		Ref		Ref		Ref	
Adequate	0.36 (0.14)	0.01	0.29 (0.14)	0.04	0.18 (0.13)	0.17	0.16 (0.13)	0.22	0.34 (0.14)	0.01	0.25 (0.14)	0.08	0.23 (0.12)	0.05
Current/prior cancer diagnosis														
No	Ref		Ref		Ref		Ref		Ref		Ref		Ref	
Yes	-0.37 (0.18)	0.05	-0.38 (0.19)	0.05	-0.18 (0.18)	0.33	-0.25 (0.17)	0.14	-0.37 (0.19)	0.05	-0.20 (0.19)	0.29	0.01 (0.16)	0.92
Being a healthcare provider														
No	Ref		Ref		Ref		Ref		Ref		Ref		Ref	
Yes	-0.02 (0.19)	0.91	-0.19 (0.19)	0.92	0.05 (0.18)	0.78	-0.03 (0.17)	0.86	0.10 (0.18)	0.58	0.04 (0.19)	0.83	-0.08 (0.16)	0.63
Leisure Score index														
≤ 24	Ref		Ref		Ref		Ref		Ref		Ref		Ref	
> 24	0.13 (0.15)	0.39	0.25 (0.15)	0.09	0.22 (0.14)	0.12	0.28 (0.13)	0.03	0.26 (0.14)	0.07	0.28 (0.15)	0.06	0.05 (0.12)	0.67

## Discussion

This is the first study investigating the population's perception regarding physical exercise in patients with cancer undergoing treatments. The POPCORN study found that more than half of respondents have a positive perception regarding the role of physical exercise in the cancer context, even if a low rate of participants think that patients are capable of exercising during anticancer treatments and find exercise participation easy.

Regarding attitude, about 60% of respondents agree about the importance and the beneficial effect of physical exercise for patients with cancer during treatments. These findings are in line with studies addressed to clinicians, which show similar results and sometimes higher rates regarding the consideration of importance (55.8-99.0%) and benefits (62.0-77.8%) of physical exercise in cancer (27, 28, 33, 35). Furthermore, patients with cancer also seem aware of the different benefits derived from physical exercise (19, 36). For instance, a systematic review including 98 studies demonstrated that patients have a positive attitude to physical exercise, and additionally, they perceive both the physiological benefits, such as improvement in fitness, strength, survival, and recurrence, in boosting energy and preventing the weight loss, as well as the psychosocial advantages, including the enhancements in relieving stress, improving mood, socialization, quality of sleep and self-esteem, related to exercise (25). Our results could be related to the low rate of physically active participants, only 28%. Indeed, not experiencing the benefits of physical exercise may be why some people believe it is not helpful for patients either.

About the safety of physical exercise, we found that about 60% of respondents disagreed or expressed a neutral opinion. Surveys conducted on clinicians and oncologists reported a higher level of confidence concerning the safety of this lifestyle intervention (29, 37). Additionally, there is a strong backbone of literature supporting the safety profile of exercise, even in the context of advanced-stage disease and bone metastasis, which are theoretically considered a greater risk of adverse events (10, 39–43). *Heywood and colleagues*, in their systematic review evaluating the safety of a physical exercise intervention, reported that among 1,088 included patients with advanced cancer, only 6, i.e., 0.55%, non-severe exercise-related adverse events, predominantly minor musculoskeletal, were recorded (41). Similarly, in the systematic review of *Weller and colleagues* focused on bone metastases, a physical exercise intervention in a controlled trial setting did not increase the risk of side effects, such as a pathological fracture or pain (44). However, of interest, in our study, we found that a significantly higher percentage of persons with a history of cancer (about 20% vs. 10%) disagree about the importance, benefits, and safety of physical exercise. Although it is difficult to explain such results, which could be associated with cancer-related issues not assessed in this survey, the risk of injury and some symptoms, such as pain and fatigue exacerbation, are sporadically described as potential issues in research analyzing the perceptions of patients (25). Whereas future investigations could deeply analyze the reasons behind the negative attitude about exercise oncology, the fact that about 4-6 out of 10 people are not still convinced of the positive and safety impact of physical exercise could lead people who have family members and/or friends with cancer to discourage them in maintaining an active lifestyle, as well as to generate an environment which is unlikely to support an active lifestyle. In this light, mandatory efforts to spread the importance of physical exercise (e.g., through social media and public health campaigns), are needed in order to lead to a true implementation in the future.

About half of the respondents of this survey believed in the supportive role of oncologists and family/friends in advising and encouraging patients to exercise. In the literature, patients themselves often report preferring to receive information from their oncologist or nurse (25), and also a percentage between 54.9% to 69.9% of healthcare providers believe that promoting physical exercise should be part of their role (44–46). However, in practice, physical exercise promotion is more complex. On one side, assessing and advising patients to increase their physical activity appears more incorporated in the routine clinical practice (29, 47), whereas the referral is still a challenge, with only 10% of lung cancer care professionals referring their patients to a dedicated exercise service (29, 48). Several barriers, such as lack of time and access to a dedicated exercise specialist (29, 30, 47), have been identified and could be overcome in different ways (e.g., by providing quick but effective advice). Indeed, in this sense, two different randomized controlled trials conducted on patients with breast or colorectal cancer reported the positive effect of oncologist advice in promoting physical activity (49, 50). On the other hand, also support from family and friends is crucial, and studies conducted on patient preferences confirm the willingness of patients to exercise with a partner (51). Social support may encourage and effectively promote physical exercise engagement of patients with cancer (23, 52), enhance emotional well-being, and at the same time, in the context of physical activity, may offer the chance to find support for coping with cancer (23). Because of the mounting wave of oncological diseases expected in the next years (53), increase awareness and knowledge about the role of social support in cancer, as well as create educational tools to inform family and friends about the benefits and safety of an active lifestyle, is essential to offering the right support to patients.

However, only 27.2% and 9.0% of the respondents in the POPCORN study agree about the capability and ease for patients with cancer of exercising during treatment, respectively. Although the precise reasons for these answers were not investigated, these findings may reflect the current misperception of patients with cancer and the anticancer treatments and could suggest that the stigma related to this disease still prevails. To reinforce this assumption, the participants estimated that less than 20% of patients are physically active. Over the years, cancer has inspired fear among the population, so much so that the term *carcinophobia* is used to characterize the anxiety and fear of developing cancer. Nevertheless, the advancements in early detection strategies, as well as in the discovery of innovative and more efficacious therapeutic approaches (e.g., target therapy and immunotherapy), have led to undeniable improvements in prognosis and quality of life for most cancer types (54), carrying cancer to become officially a chronic disease. Together with these enhancements, the research regarding exercise oncology has registered exponential growth, strongly demonstrating the feasibility for patients with cancer to participate in a tailored physical exercise program, independently from the type and stage of disease (12, 39–41) and also providing specific indications for exercise specialists to tailored and personalized the exercise program to the different conditions (5, 55). The perception of patients is positive on this point. Across the studies, a range of 47-90% of patients feel able to perform physical activity, and 78-95% express interest in participating in a dedicated program (51).

In our study, among the socio-demographic characteristics, being physically active is linked to a better perception of capability. Although the exact reasons for these associations are not understood, it is possible that engaging in physical activity could make people more aware of the possibility of adapting physical exercise to various conditions, including cancer. Additionally, we found that geographic and socioeconomic factors also played a role in shaping perceptions. Those living in the Central South of Italy and respondents who reported financial adequacy were more likely to agree that patients are capable of exercising and that exercise is beneficial. On the other hand, respondents in northern Italy and those with financial difficulties tended to hold more neutral opinions on these topics, possibly reflecting regional and economic disparities in access to information or support for exercise during cancer treatment. Another issue that may influence the results of our study could be the exercise setting (i.e., supervised vs. home-based programs). The literature highlights the pros and cons of supervised (e.g., constant supervision and monitoring by experts, more tailoring exercise) and home-based (e.g., time and cost saving, avoiding the discomfort of exercising with others) programs (56–59) and supports the safety and effectiveness in improving the functional capacity and quality of life of both (60, 61). Nevertheless, it could be possible that the proposed setting influences population perception. For instance, a supervised program could appear to be a safer strategy, while a home-based program could be more accessible for patients and, therefore, easier to implement. In the future, all these speculations could be explored, and it could also be investigated if the perception of patients’ capability of exercising depends on disease-related factors (e.g., type of cancer, stage of disease, specific treatments). In any case, the current results about patients’ capability to perform physical exercise may indicate that efforts should be made to overcome the stigma related to exercise and cancer (e.g., spreading available programs for patients or organizing dedicated days to support the feasibility of physical exercise in this context).

The current study has strengths and limitations that should be noted. Firstly, the method of survey diffusion, i.e., social media and the use of one social network, could have potentially led to selection bias. Nevertheless, Facebook, as reported in the methodology, can offer several advantages as well as, in the literature, it is the most widely used social media with published instructions for conducting studies on this platform (62, 63). Although a relatively younger population has responded to our survey, the other socio-demographic characteristics, including gender, occupational status, and physical activity levels, are comparable with the Italian situation, making our sample representative (64). Another source of bias could be related to the fact that our questionnaire did not include other specific information, such as being a patient caregiver, the type of cancer in the case of the patient, and the reasons for disagreement in the perception items, thus limiting the ability to explore the association between these characteristics and perceptions. However, we developed and adapted a quick questionnaire, which enabled us to collect a large amount of data without burdening the respondents. The POPCORN study represents the first investigation exploring the population perception of physical exercise in oncology among the general population, and to our knowledge, this is the first also across chronic non-communicable diseases, which has permitted provide useful data to plan future cues to action to increase the awareness of physical exercise in patients with cancer. If this study could be considered the first step in exploring this issue, in the future, the current survey could be expanded to other countries in order to capture perspectives and differences among the populations and societies. Collaborations with international researchers and advocacy groups could further facilitate its dissemination across regions and enhance sample diversity. Customizing the survey to reflect regional differences in healthcare systems, exercise practices, and cancer care policies could also provide more nuanced insights into the global landscape of exercise oncology.

In conclusion, the majority of the general population agrees about the importance and beneficial effect of physical exercise for patients with cancer, about half perceive the safety of this intervention and the potential positive impact of oncologists and family/friend support. However, the perception of the patient’s capability and ease to engage in physical exercise is still not recognized. Increasing the awareness among the population about the benefits, safety, and feasibility of physical exercise in this context may provide an important basis for supporting patients to stay active throughout their disease journey. While the present study provides a descriptive overview, it lays the groundwork for future research and interventions. Building on these findings, future work could deeply examine the link between exercise perceptions, implementation strategies, and patient outcomes, ultimately contributing to more informed and effective exercise oncology practices.

## Data Availability

The original contributions presented in the study are included in the article/**Supplementary Material**. Further inquiries can be directed to the corresponding author.

## References

[1] Vrinten C van Jaarsveld CHM Waller J von Wagner C Wardle J. The structure and demographic correlates of cancer fear. BMC Cancer. (2014) 14:597. doi: 10.1186/1471-2407-14-597 25129323 PMC4148526

[2] Vrinten C Wardle J Marlow LA. Cancer fear and fatalism among ethnic minority women in the United Kingdom. Br J Cancer. (2016) 114:597–604. doi: 10.1038/bjc.2016.15 26867159 PMC4782206

[3] Daher M. Cultural beliefs and values in cancer patients. Ann Oncol. (2012) 23 Suppl 3:66–9. doi: 10.1093/annonc/mds091 22628419

[4] Vrinten C McGregor LM Heinrich M von Wagner C Waller J Wardle J. What do people fear about cancer? A systematic review and meta-synthesis of cancer fears in the general population. Psychooncology. (2017) 26:1070–9. doi: 10.1002/pon.v26.8 PMC5573953 27643482

[5] Campbell KL Winters-Stone KM Wiskemann J May AM Schwartz AL Courneya KS. Exercise guidelines for cancer survivors: consensus statement from international multidisciplinary roundtable. Med Sci Sports Exerc. (2019) 51:2375–90. doi: 10.1249/MSS.0000000000002116 PMC8576825 31626055

[6] Caspersen C.J. PKE Christenson GM. Physical activity, exercise, and physical fitness: definitions and distinctions for health-related research. Public Health Rep. (1985) 100:126–31. PMC1424733 3920711

[7] Friedenreich CM Stone CR Cheung WY Hayes SCv. Physical activity and mortality in cancer survivors: A systematic review and meta-analysis. JNCI Cancer Spectr. (2020) 4:pkz080. doi: 10.1093/jncics/pkz080 32337494 PMC7050161

[8] Verheijden RJ Ballester AC Smit KC van Eijs MJM Bruijnen CP van Lindert ASR. Physical activity and checkpoint inhibition: association with toxicity and survival. J Natl Cancer Inst. (2023) 116(4):573–9. doi: 10.1093/jnci/djad245 PMC10995850 38001030

[9] Scott JM Zabor EC Schwitzer E Koelwyn GJ Adams SC Nilsen TS. Efficacy of exercise therapy on cardiorespiratory fitness in patients with cancer: A systematic review and meta-analysis. J Clin Oncol. (2018) 36:2297–305. doi: 10.1200/JCO.2017.77.5809 PMC6804903 29894274

[10] Avancini A Sperduti I Borsati A Ferri T Belluomini L Insolda J. Effect of exercise on functional capacity in patients with advanced cancer: A meta-analysis of randomized controlled trials. Crit Rev Oncol Hematol. (2022) 175:103726. doi: 10.1016/j.critrevonc.2022.103726 35659975

[11] Koeppel M Mathis K Schmitz KH Wiskemann J. Muscle hypertrophy in cancer patients and survivors via strength training. A meta-analysis and meta-regression. Crit Rev Oncol Hematol. (2021) 163:103371. doi: 10.1016/j.critrevonc.2021.103371 34062243

[12] Avancini A Borsati A Baldo E Ciurnelli C Trestini I Tregnago D. A feasibility study investigating an exercise program in metastatic cancer based on the patient-preferred delivery mode. Oncologist. (2024) 29(6):e828–36. doi: 10.1093/oncolo/oyae002 PMC11144977 38206849

[13] Law CYJ Yu THJ Chen T. Effectiveness of aerobic and resistance exercise in cancer survivors with depression: A systematic review and meta-analysis of randomized controlled trials. J Psychosom Res. (2023) 173:111470. doi: 10.1016/j.jpsychores.2023.111470 37643561

[14] Avancini A Borsati A Trestini I Tregnago D Belluomini L Sposito M. Exploring the feasibility of a combined exercise program for patients with advanced lung or pancreatic cancer. Asia Pac J Oncol Nurs. (2023) 10:100298. doi: 10.1016/j.apjon.2023.100298 38197044 PMC10772206

[15] Ligibel JA Bohlke K May AM Clinton SK Demark-Wahnefried W Gilchrist SC. Exercise, diet, and weight management during cancer treatment: ASCO guideline. J Clin Oncol. (2022) 40:2491–507. doi: 10.1200/JCO.22.00687 35576506

[16] Rock CL Thomson CA Sullivan KR Howe CL Kushi LH Caan BJ. American Cancer Society nutrition and physical activity guideline for cancer survivors. CA Cancer J Clin. (2022) 72:230–62. doi: 10.3322/caac.21719 35294043

[17] Avancini A Trestini I Tregnago D Belluomini L Sposito M Insolda J. Willingness, preferences, barriers, and facilitators of a multimodal supportive care intervention including exercise, nutritional and psychological approach in patients with cancer: a cross-sectional study. J Cancer Res Clin Oncol. (2023) 149:3435–45. doi: 10.1007/s00432-022-04232-6 PMC10314831 35943598

[18] Glanz K Bishop DB. The role of behavioral science theory in development and implementation of public health interventions. Annu Rev Public Health. (2010) 31:399–418. doi: 10.1146/annurev.publhealth.012809.103604 20070207

[19] Andersen C Adamsen L Damhus CS Pill K Missel M Jarden M. Qualitative exploration of the perceptions of exercise in patients with cancer initiated during chemotherapy: a meta-synthesis. BMJ Open. (2023) 13:e074266. doi: 10.1136/bmjopen-2023-074266 PMC10729187 38086582

[20] Hardcastle SJ Maxwell-Smith C Kamarova S Lamb S Millar L Cohen PA. Factors influencing non-participation in an exercise program and attitudes towards physical activity amongst cancer survivors. Support Care Cancer. (2018) 26:1289–95. doi: 10.1007/s00520-017-3952-9 29090387

[21] Ranes M Wiestad TH Thormodsen I Arving C. Determinants of exercise adherence and maintenance for cancer survivors: Implementation of a community-based group exercise program. A qualitative feasibility study. PEC Innov. (2022) 1:100088. doi: 10.1016/j.pecinn.2022.100088 37213720 PMC10194213

[22] McDonough MH Beselt LJ Daun JT Shank J Culos-Reed SN Kronlund LK. The role of social support in physical activity for cancer survivors: A systematic review. Psychooncology. (2019) 28:1945–58. doi: 10.1002/pon.v28.10 31278800

[23] McDonough MH Beselt LJ Kronlund LJ Albinati NK Daun JT Trudeau MS. Social support and physical activity for cancer survivors: a qualitative review and meta-study. J Cancer Surviv. (2021) 15:713–28. doi: 10.1007/s11764-020-00963-y 33128705

[24] Ulrich GR Callan S Ranby KW. Beliefs and interests in physical activity programs of cancer survivors and their romantic partners. J Cancer Surviv. (2023) 17:160–73. doi: 10.1007/s11764-021-00996-x PMC7886842 33595753

[25] Elshahat S Treanor C Donnelly M. Factors influencing physical activity participation among people living with or beyond cancer: a systematic scoping review. Int J Behav Nutr Phys Act. (2021) 18:50. doi: 10.1186/s12966-021-01116-9 33823832 PMC8025326

[26] Borsati A. MA Ducoli V Dodi A Belluomini L Schena F Milella M. A qualitative study exploring the experiences and perspectives of patients with cancer attending a 12-week exercise program. Sport Sci Health. (2023) 19:993–1001. doi: 10.1007/s11332-023-01055-x

[27] Afaya A Anaba EA Bam V Afaya RA Yahaya A Seidu A. Socio-cultural beliefs and perceptions influencing diagnosis and treatment of breast cancer among women in Ghana: a systematic review. BMC Womens Health. (2024) 24:288. doi: 10.1186/s12905-024-03106-y 38745160 PMC11092234

[28] Avancini A D'Amico F Tregnago D Trestini I Belluomini L Vincenzi S. Nurses' perspectives on physical activity promotion in cancer patients: A qualitative research. Eur J Oncol Nurs. (2021) 55:102061. doi: 10.1016/j.ejon.2021.102061 34763207

[29] Pilotto S Avancini A Menis J Sperduti I Levra MG Berghmans T. Exercise in lung Cancer, the healthcare providers opinion (E.C.H.O.): Results of the EORTC lung cancer Group (LCG) survey. Lung Cancer. (2022) 169:94–101. doi: 10.1016/j.lungcan.2022.05.009 35691097

[30] Alderman G Semple S Cesnik R Toohey K. Health care professionals' Knowledge and attitudes toward physical activity in cancer patients: A systematic review. Semin Oncol Nurs. (2020) 36:151070. doi: 10.1016/j.soncn.2020.151070 33010981

[31] Simon K. Digital 2023: Italy. DataReportal (2023). Available online at: https://datareportal.com/reports/digital-2023-italy (Accessed November 28, 2024).

[32] Hargittai E. Potential biases in big data: omitted voices on social media. Soc Sci Comput Rev. (2018) 38:10–24. doi: 10.1177/0894439318788322

[33] Grow A. Addressing public health emergencies via facebook surveys: advantages, challenges, and practical considerations. J Med Internet Res. (2020) 22:e20653. doi: 10.2196/20653 33284782 PMC7744148

[34] Whitaker C Stevelink S Fear N. The use of facebook in recruiting participants for health research purposes: A systematic review. J Med Internet Res. (2017) 19:e290. doi: 10.2196/jmir.7071 28851679 PMC5594255

[35] von Elm E Altman DG Egger M Pocock SJ Gotzsche PC Vandenbroucke JP. The Strengthening the Reporting of Observational Studies in Epidemiology (STROBE) statement: guidelines for reporting observational studies. J Clin Epidemiol. (2008) 61:344–9. doi: 10.1016/j.jclinepi.2007.11.008 18313558

[36] Eysenbach G. Improving the quality of Web surveys: the Checklist for Reporting Results of Internet E-Surveys (CHERRIES). J Med Internet Res. (2004) 6:e34. doi: 10.2196/jmir.6.3.e34 15471760 PMC1550605

[37] Jones LW Courneya KS Peddle C Mackey JR. Oncologists' opinions towards recommending exercise to patients with cancer: a Canadian national survey. Support Care Cancer. (2005) 13:929–37. doi: 10.1007/s00520-005-0805-8 15809835

[38] Amireault S Godin G Lacombe J Sabiston CM. The use of the Godin-Shephard Leisure-Time Physical Activity Questionnaire in oncology research: a systematic review. BMC Med Res Methodol. (2015) 15:60. doi: 10.1186/s12874-015-0045-7 26264621 PMC4542103

[39] Singh B Spence RR Steele ML Sandler CX Peake JM Hayes SC. A systematic review and meta-analysis of the safety, feasibility, and effect of exercise in women with stage II+ Breast cancer. Arch Phys Med Rehabil. (2018) 99:2621–36. doi: 10.1016/j.apmr.2018.03.026 29730319

[40] Cave J Paschalis A Huang CY West M Copson E Jack S. A systematic review of the safety and efficacy of aerobic exercise during cytotoxic chemotherapy treatment. Supportive Care Cancer. (2018) 26:3337–51. doi: 10.1007/s00520-018-4295-x 29936624

[41] Heywood R McCarthy AL Skinner TL. Safety and feasibility of exercise interventions in patients with advanced cancer: a systematic review. Supportive Care Cancer. (2017) 25:3031–50. doi: 10.1007/s00520-017-3827-0 28741176

[42] Singh B Hayes SC Spence RR Steele ML Millet G Gergele L. Exercise and colorectal cancer: a systematic review and meta-analysis of exercise safety, feasibility and effectiveness. Int J Behav Nutr Phys Activity. (2020) 17:122. doi: 10.1186/s12966-020-01021-7 PMC7513291 32972439

[43] Avancini A Benato G Borsati A Oliviero L Belluomini L Sposito M. Exercise and bone health in cancer: enemy or ally? Cancers (Basel). (2022) 14. doi: 10.3390/cancers14246078 PMC9776461 36551564

[44] Weller S Hart NH Bolam KA Mansfield S Santa Mina D Winters-Stone KM. Exercise for individuals with bone metastases: A systematic review. Crit Rev Oncology/Hematology. (2021) 166:103433. doi: 10.1016/j.critrevonc.2021.103433 34358650

[45] Karvinen KH McGourty S Parent T Walker PR. Physical activity promotion among oncology nurses. Cancer Nurs. (2012) 35:E41–8. doi: 10.1097/NCC.0b013e31822d9081 21946904

[46] Tsiouris A Ungar N Haussmann A Sieverding M Steindorf K Wiskemann J. Health care professionals' Perception of contraindications for physical activity during cancer treatment. Front Oncol. (2018) 8:98. doi: 10.3389/fonc.2018.00098 29670858 PMC5894008

[47] Ligibel JA Jones LW Brewster AM Clinton SK Korde LA Oeffinger KC. Oncologists' Attitudes and practice of addressing diet, physical activity, and weight management with patients with cancer: findings of an ASCO survey of the oncology workforce. J Oncol Pract. (2019) 15:e520–8. doi: 10.1200/JOP.19.00124 PMC6827390 31095436

[48] Hardcastle SJ Kane R Chivers P Hince D Dean A Higgs D. Knowledge, attitudes, and practice of oncologists and oncology health care providers in promoting physical activity to cancer survivors: an international survey. Support Care Cancer. (2018) 26:3711–9. doi: 10.1007/s00520-018-4230-1 29740694

[49] Jones LW Courneya KS Fairey AS Mackey JR. Effects of an oncologist's recommendation to exercise on self-reported exercise behavior in newly diagnosed breast cancer survivors: a single-blind, randomized controlled trial. Ann Behav Med. (2004) 28:105–13. doi: 10.1207/s15324796abm2802_5 15454357

[50] Park J Lee J Oh M Park H Chae J Kim D. The effect of oncologists' exercise recommendations on the level of exercise and quality of life in survivors of breast and colorectal cancer: A randomized controlled trial. Cancer. (2015) 121:2740–8. doi: 10.1002/cncr.v121.16 PMC5025035 25965782

[51] Wong JN McAuley E Trinh L. Physical activity programming and counseling preferences among cancer survivors: a systematic review. Int J Behav Nutr Phys Act. (2018) 15:48. doi: 10.1186/s12966-018-0680-6 29879993 PMC5992647

[52] Ungar N Wiskemann J Weißmann M Knoll A Steindorf K Sieverding M. Social support and social control in the context of cancer patients' exercise: A pilot study. Health Psychol Open. (2016) 3:2055102916680991. doi: 10.1177/2055102916680991 28815053 PMC5546267

[53] Iarc. Cancer Tomorrow. Available online at: https://gco.iarc.fr/tomorrow/en (Accessed April 15, 2024).

[54] Dal Maso L Panato C Guzzinati S Serraino D Francisci S Botta L. Prognosis and cure of long-term cancer survivors: A population-based estimation. Cancer Med. (2019) 8:4497–507. doi: 10.1002/cam4.2276 PMC6675712 31207165

[55] Campbell KL Cormie P Weller S Alibhai SMH Bolam KA Campbell A. Exercise recommendation for people with bone metastases: expert consensus for health care providers and exercise professionals. JCO Oncol Pract. (2022) 18:e697–709. doi: 10.1200/OP.21.00454 PMC9810134 34990293

[56] Hardcastle SJ Cohen PA. Effective physical activity promotion to survivors of cancer is likely to be home based and to require oncologist participation. J Clin Oncol. (2017) 35:3635–37. doi: 10.1200/jco.2017.74.6032 28915086

[57] Lopez C Jones J Alibhai SMH Santa Mina D. What is the "Home" in home-based exercise? The need to define independent exercise for survivors of cancer. J Clin Oncol. (2018) 36:926–7. doi: 10.1200/JCO.2017.76.4365 29373096

[58] Newton RU Taaffe DR Chambers SK Spry N Galvão DA. Effective exercise interventions for patients and survivors of cancer should be supervised, targeted, and prescribed with referrals from oncologists and general physicians. J Clin Oncol. (2018) 36:927–8. doi: 10.1200/JCO.2017.76.7400 29373097

[59] Adams SC Iyengar NM Scott JM Jones LW. Exercise implementation in oncology: one size does not fit all. J Clin Oncol. (2018) 36:925–6. doi: 10.1200/JCO.2017.76.2906 29373093

[60] Pelosi AC Rostirola GC Pereira JS Silva KC Fontanari MER Oliveira MSP. Remote and unsupervised exercise strategies for improving the physical activity of colorectal cancer patients: A meta-analysis. Healthcare (Basel). (2023) 11. doi: 10.3390/healthcare11050723 PMC10000866 36900728

[61] Kraemer MB Priolli DG Reis IGM Pelosi AC Garbuio ALP Messias LHD. Home-based, supervised, and mixed exercise intervention on functional capacity and quality of life of colorectal cancer patients: a meta-analysis. Sci Rep. (2022) 12:2471. doi: 10.1038/s41598-022-06165-z 35169171 PMC8847564

[62] Kosinski M Matz SC Gosling SD Popov V Stillwell D. Facebook as a research tool for the social sciences: Opportunities, challenges, ethical considerations, and practical guidelines. Am Psychol. (2015) 70:543–56. doi: 10.1037/a0039210 26348336

[63] Pedersen ER Kurz J. Using facebook for health-related research study recruitment and program delivery. Curr Opin Psychol. (2016) 9:38–43. doi: 10.1016/j.copsyc.2015.09.011 26726313 PMC4697271

[64] Istat. Rapporto Annuale 2024, la situazione del paese (2024). Available online at: https://www.istat.it/it/files/2024/05/Sintesi-Rapporto-Annuale-2024.pdf (Accessed April 15, 2024).

