# Supplementary material for: A cross-sectional study exploring the perception of exercise oncology in the Italian population
Source: Front Oncol. 2025 Jan 13;14:1430083. doi: 10.3389/fonc.2024.1430083 (PMC11769818; doi:10.3389/fonc.2024.1430083)
Supplement: Supplementary file 1 [file Table1.docx]

**Supplementary Material**

**Table S1**. Perception of exercise as beneficial according to participants’ characteristics

**Table S2**. Perception of exercise as important according to participants' characteristics

**Table S3**. Perception of exercise as safe according to participants' characteristics

**Table S4**. Perception of patients’ capability to perform exercise according to participants' characteristics

**Table S5.** Perception about the role of oncologists in advising patients to perform exercise according to participants' characteristics

**Table S6**. Perception about the role of family/friends in encouraging patients to perform exercise according to participants' characteristics

**Table S7**. Perception of ease for patients to perform exercise according to participants' characteristics

**Table S8.** Non healthcare providers’ perception regarding physical activity in patients with cancer

**Table S9.** The percentage of active patients with cancer during treatment according to non-healthcare providers

**Table S10.** Multivariable regression of associations of non-healthcare providers' characteristics with their perception regarding exercise in cancer

**Table S11**. Perception of exercise as beneficial according to non-healthcare providers' characteristics

**Table S12**. Perception of exercise as important according to non-healthcare providers' characteristics

**Table S13**. Perception of exercise as safe according to non-healthcare providers' characteristics

**Table S14**. Perception of patients’ capability to perform exercise according to non-healthcare providers' characteristics

**Table S15.** Perception about the role of oncologists in advising patients to perform exercise according to non-healthcare providers' characteristics

**Table S16**. Perception about the role of family/friends in encouraging patients to perform exercise according to non-healthcare providers' characteristics

**Table S17**. Perception of ease for patients to perform exercise according to non-healthcare providers' characteristics

**Table S1**. Perception of exercise as beneficial according to participants’ characteristics

|  | **Disagree, N (%)** | **Neutral, N (%)** | **Agree, N (%)** | **p-value** |
| --- | --- | --- | --- | --- |
| Gender |  |  |  |  |
| Male | 28 (9.7) | 94 (32.4) | 168 (57.9) | 0.007 |
| Female | 77 (14.2) | 125 (23.1) | 339 (62.7) |  |
| Age |  |  |  |  |
| ≤ 39 | 40 (9.5) | 105 (25.1) | 274 (65.4) | 0.008 |
| > 39 | 65 (15.8) | 114 (27.7) | 233 (56.6) |  |
| Region |  |  |  |  |
| North | 85 (12.1) | 197 (28.1) | 418 (59.7) | 0.027 |
| Central-South | 18 (14.9) | 20 (16.5) | 83 (68.6) |  |
| Occupational status |  |  |  |  |
| Employed | 77 (13.0) | 152 (25.6) | 364 (61.4) | 0.77 |
| Not employed | 28 (11.8) | 66 (27.8) | 143 (60.3) |  |
| Financial difficulties |  |  |  |  |
| Yes | 43 (16.5) | 65 (24.9) | 153 (58.6) | 0.080 |
| No | 62 (10.9) | 153 (26.9) | 354 (62.2) |  |
| Current/prior cancer diagnosis |  |  |  |  |
| No | 74 (10.6) | 198 (28.3) | 428 (61.1) | <0.0001 |
| Yes | 31 (23.8) | 20 (15.4) | 79 (60.8) |  |
| Healthcare provider |  |  |  |  |
| Yes | 19 (17.3) | 17 (15.5) | 74 (67.3) | 0.016 |
| No | 85 (12.0) | 196 (27.6) | 428 (60.4) |  |
| LSI |  |  |  |  |
| < 24 | 79 (13.3) | 164 (27.6) | 351 (59.1) | 0.17 |
| ≥ 24 | 25 (10.6) | 55 (23.3) | 156 (66.1) |  |

**Table S2**. Perception of exercise as important according to participants' characteristics

|  | **Disagree, N (%)** | **Neutral, N (%)** | **Agree, N (%)** | **p-value** |
| --- | --- | --- | --- | --- |
| Gender |  |  |  |  |
| Male | 29 (10.0) | 91 (31.5) | 169 (58.5) | 0.003 |
| Female | 80 (14.7) | 117 (21.5) | 346 (63.7) |  |
| Age |  |  |  |  |
| ≤ 39 | 44 (10.5) | 97 (23.2) | 278 (66.3) | 0.016 |
| > 39 | 65 (15.7) | 111 (26.9) | 237 (57.4) |  |
| Region |  |  |  |  |
| North | 89 (12.7) | 183 (26.1) | 428 (61.1) | 0.22 |
| Central-South | 18 (14.8) | 23 (18.9) | 81 (66.4) |  |
| Occupational status |  |  |  |  |
| Employed | 79 (13.3) | 146 (24.7) | 367 (62.0) | 0.94 |
| Not employed | 30 (12.6) | 61 (25.5) | 148 (61.9) |  |
| Financial difficulties |  |  |  |  |
| Yes | 46 (17.6) | 57 (21.8) | 158 (60.5) | 0.024 |
| No | 63 (11.1) | 150 (26.3) | 357 (62.6) |  |
| Current/prior cancer diagnosis |  |  |  |  |
| No | 77 (11.0) | 187 (26.8) | 435 (62.2) | <0.0001 |
| Yes | 32 (24.2) | 20 (15.2) | 80 (60.6) |  |
| Healthcare provider |  |  |  |  |
| Yes | 19 (17.1) | 19 (17.1) | 73 (65.8) | 0.074 |
| No | 87 (12.3) | 186 (26.2) | 436 (61.5) |  |
| LSI |  |  |  |  |
| < 24 | 82 (13.8) | 161 (27.1) | 351 (59.1) | 0.024 |
| ≥ 24 | 26 (11.0) | 47 (19.8) | 164 (69.2) |  |

**Table S3**. Perception of exercise as safe according to participants' characteristics

|  | **Disagree, N (%)** | **Neutral, N (%)** | **Agree, N (%)** | **p-value** |
| --- | --- | --- | --- | --- |
| Gender |  |  |  |  |
| Male | 32 (11.0) | 155 (53.3) | 104 (35.7) | 0.013 |
| Female | 78 (14.4) | 231 (42.6) | 233 (43.0) |  |
| Age |  |  |  |  |
| ≤ 39 | 44 (10.5) | 209 (49.9) | 166 (39.6) | 0.029 |
| > 39 | 66 (15.9) | 177 (42.8) | 171 (41.3) |  |
| Region |  |  |  |  |
| North | 88 (12.6) | 339 (48.4) | 274 (39.1) | 0.041 |
| Central-South | 20 (16.4) | 44 (36.1) | 58 (47.5) |  |
| Occupational status |  |  |  |  |
| Employed | 79 (13.3) | 270 (45.4) | 246 (41.3) | 0.69 |
| Not employed | 31 (13.1) | 115 (48.5) | 91 (38.4) |  |
| Financial difficulties |  |  |  |  |
| Yes | 47 (17.9) | 108 (41.2) | 107 (40.8) | 0.014 |
| No | 63 (11.1) | 277 (48.6) | 230 (40.4) |  |
| Current/prior cancer diagnosis |  |  |  |  |
| No | 78 (11.1) | 348 (49.7) | 274 (39.1) | <0.0001 |
| Yes | 32 (24.2) | 37 (28.0) | 63 (47.7) |  |
| Healthcare provider |  |  |  |  |
| Yes | 20 (18.0) | 35 (31.5) | 56 (50.5) | 0.004 |
| No | 87 (12.3) | 343 (48.3) | 280 (39.4) |  |
| LSI |  |  |  |  |
| < 24 | 84 (14.1) | 284 (47.7) | 228 (38.3) | 0.08 |
| ≥ 24 | 25 (10.6) | 102 (43.2) | 109 (46.2) |  |

**Table S4**. Perception of patients’ capability to perform exercise according to participants' characteristics

|  | **Disagree, N (%)** | **Neutral, N (%)** | **Agree, N (%)** | **p-value** |
| --- | --- | --- | --- | --- |
| Gender |  |  |  |  |
| Male | 40 (13.7) | 180 (61.6) | 72 (24.7) | 0.30 |
| Female | 82 (15.1) | 305 (56.2) | 156 (28.7) |  |
| Age |  |  |  |  |
| ≤ 39 | 55 (13.1) | 254 (60.6) | 110 (26.3) | 0.28 |
| > 39 | 67 (16.1) | 231 (55.5) | 118 (28.4) |  |
| Region |  |  |  |  |
| North | 102 (14.5) | 421 (60.0) | 179 (25.5) | 0.018 |
| Central-South | 18 (14.6) | 59 (48.0) | 46 (37.4) |  |
| Occupational status |  |  |  |  |
| Employed | 91 (15.3) | 343 (57.6) | 161 (27.1) | 0.69 |
| Not employed | 31 (13.0) | 141 (59.0) | 67 (28.0) |  |
| Financial difficulties |  |  |  |  |
| Yes | 53 (20.2) | 131 (49.8) | 79 (30.0) | 0.001 |
| No | 69 (12.1) | 353 (61.8) | 149 (26.1) |  |
| Current/prior cancer diagnosis |  |  |  |  |
| No | 95 (13.5) | 415 (59.0) | 193 (27.5) | 0.10 |
| Yes | 27 (20.6) | 69 (52.7) | 35 (26.7) |  |
| Healthcare provider |  |  |  |  |
| Yes | 20 (18.0) | 58 (52.3) | 33 (29.7) | 0.42 |
| No | 102 (14.3) | 416 (58.4) | 194 (27.2) |  |
| LSI |  |  |  |  |
| < 24 | 90 (15.1) | 355 (59.6) | 151 (25.3) | 0.12 |
| ≥ 24 | 31 (13.0) | 130 (54.6) | 77 (32.4) |  |

**Table S5.** Perception about the role of oncologists in advising patients to perform exercise according to participants' characteristics

|  | **Disagree, N (%)** | **Neutral, N (%)** | **Agree, N (%)** | **p-value** |
| --- | --- | --- | --- | --- |
| Gender |  |  |  |  |
| Male | 30 (10.3) | 116 (39.7) | 146 (50.0) | 0.046 |
| Female | 80 (14.7) | 176 (32.3) | 289 (53.0) |  |
| Age |  |  |  |  |
| ≤ 39 | 40 (9.5) | 156 (37.2) | 223 (53.2) | 0.007 |
| > 39 | 70 (16.7) | 136 (32.5) | 212 (50.7) |  |
| Region |  |  |  |  |
| North | 89 (12.7) | 259 (36.8) | 355 (50.5) | 0.039 |
| Central-South | 19 (15.3) | 31 (25.0) | 74 (59.7) |  |
| Occupational status |  |  |  |  |
| Employed | 75 (12.6) | 211 (35.3) | 311 (52.1) | 0.69 |
| Not employed | 35 (14.6) | 80 (33.5) | 124 (51.9) |  |
| Financial difficulties |  |  |  |  |
| Yes | 47 (17.7) | 88 (33.2) | 130 (49.1) | 0.028 |
| No | 63 (11.0) | 203 (35.6) | 305 (53.4) |  |
| Current/prior cancer diagnosis |  |  |  |  |
| No | 80 (11.4) | 255 (36.2) | 369 (52.4) | 0.001 |
| Yes | 30 (22.7) | 36 (27.3) | 66 (50.0) |  |
| Healthcare provider |  |  |  |  |
| Yes | 20 (18.0) | 23 (20.7) | 68 (61.3) | 0.004 |
| No | 90 (12.6) | 262 (36.7) | 362 (50.7) |  |
| LSI |  |  |  |  |
| < 24 | 83 (13.9) | 221 (36.9) | 295 (49.2) | 0.037 |
| ≥ 24 | 26 (11.0) | 71 (30.0) | 140 (59.1) |  |

**Table S6**. Perception about the role of family/friends in encouraging patients to perform exercise according to participants' characteristics

|  | **Disagree, N (%)** | **Neutral, N (%)** | **Agree, N (%)** | **p-value** |
| --- | --- | --- | --- | --- |
| Gender |  |  |  |  |
| Male | 33 (11.3) | 112 (38.4) | 147 (50.3) | 0.06 |
| Female | 86 (15.8) | 171 (31.4) | 287 (52.8) |  |
| Age |  |  |  |  |
| ≤ 39 | 44 (10.5) | 146 (34.8) | 229 (54.7) | 0.008 |
| > 39 | 75 (18.0) | 137 (32.9) | 205 (49.2) |  |
| Region |  |  |  |  |
| North | 94 (13.4) | 247 (35.2) | 361 (51.4) | 0.11 |
| Central-South | 23 (18.5) | 33 (26.6) | 68 (54.8) |  |
| Occupational status |  |  |  |  |
| Employed | 80 (13.4) | 205 (34.5) | 310 (52.1) | 0.54 |
| Not employed | 39 (16.3) | 77 (32.1) | 124 (51.7) |  |
| Financial difficulties |  |  |  |  |
| Yes | 51 (19.2) | 84 (31.7) | 130 (49.1) | 0.019 |
| No | 68 (11.9) | 198 (34.7) | 304 (53.3) |  |
| Current/prior cancer diagnosis |  |  |  |  |
| No | 89 (12.6) | 250 (35.5) | 365 (51.8) | 0.002 |
| Yes | 30 (22.9) | 32 (24.4) | 69 (52.7) |  |
| Healthcare provider |  |  |  |  |
| Yes | 22 (19.8) | 22 (19.8) | 67 (60.4) | 0.003 |
| No | 97 (13.6) | 254 (35.6) | 362 (50.8) |  |
| LSI |  |  |  |  |
| < 24 | 91 (15.2) | 216 (36.2) | 290 (48.6) | 0.008 |
| ≥ 24 | 27 (11.3) | 67 (28.2) | 144 (60.5) |  |

**Table S7**. Perception of ease for patients to perform exercise according to participants' characteristics

|  | Disagree, N (%) | Neutral, N (%) | Agree, N (%) | p-value |
| --- | --- | --- | --- | --- |
| Gender |  |  |  |  |
| Male | 84 (29.1) | 183 (63.3) | 22 (7.6) | 0.53 |
| Female | 163 (30.0) | 328 (60.3) | 53 (9.7) |  |
| Age |  |  |  |  |
| ≤ 39 | 136 (32.6) | 247 (59.2) | 34 (8.2) | 0.15 |
| > 39 | 111 (26.7) | 264 (63.5) | 41 (9.9) |  |
| Region |  |  |  |  |
| North | 212 (30.3) | 431 (61.7) | 56 (8.0) | 0.05 |
| Central-South | 31 (25.0) | 75 (60.5) | 18 (14.5) |  |
| Occupational status |  |  |  |  |
| Employed | 181 (30.5) | 356 (59.9) | 57 (9.6) | 0.40 |
| Not employed | 66 (27.7) | 154 (64.7) | 18 (7.6) |  |
| Financial difficulties |  |  |  |  |
| Yes | 92 (34.8) | 150 (56.8) | 22 (8.3) | 0.08 |
| No | 155 (27.3) | 360 (63.4) | 53 (9.3) |  |
| Current/prior cancer diagnosis |  |  |  |  |
| No | 210 (30.0) | 425 (60.7) | 65 (9.3) | 0.69 |
| Yes | 37 (28.0) | 85 (64.4) | 10 (7.6) |  |
| Healthcare provider |  |  |  |  |
| Yes | 33 (29.7) | 69 (62.2) | 9 (8.1) | 0.92 |
| No | 209 (29.4) | 435 (61.3) | 66 (9.3) |  |
| LSI |  |  |  |  |
| < 24 | 179 (30.1) | 367 (61.7) | 49 (8.2) | 0.44 |
| ≥ 24 | 67 (28.3) | 144 (60.8) | 26 (11.0) |  |

**Table S8.** Non healthcare providers’ perception regarding physical activity in patients with cancer

|  | **Agree, N (%)** | **Neutral, N (%)** | **Disagree, N (%)** |
| --- | --- | --- | --- |
| In my opinion, exercise is beneficial in patients during treatment | 428 (58.9) | 196 (27.6) | 85 (12.0) |
| In my opinion, exercise is important for patients during treatment | 436 (61.5) | 186 (26.2) | 87 (12.3) |
| In my opinion, exercise is safe for patients during treatment | 280 (39.4) | 343 (48.3) | 87 (12.3) |
| In my opinion, oncologists should advise patients to exercise during treatment | 362 (50.7) | 262 (36.7) | 90 (12.6) |
| In my opinion, family or friends should encourage patients to exercise during treatment | 362 (50.8) | 254 (35.6) | 97 (13.6) |
| In my opinion, patients are capable to exercise during treatment | 194 (27.2) | 416 (58.4) | 102 (14.3) |
| In my opinion, for patients is easy to exercise during treatment | 66 (9.3) | 435 (61.3) | 209 (29.4) |

**Table S9.** The percentage of active patients with cancer during treatment according to non-healthcare providers

| **Percentage of active patients** | **Responders (%)** |
| --- | --- |
| <20% | 62.6 |
| 20-40% | 31.9 |
| 40-60% | 4.5 |
| 60-80% | 0.6 |
| >80% | 0.6 |

|  | **Exercise is beneficial** | | **Exercise is important** | | **Exercise is safe** | | **Patients are able to exercise** | | **Oncologists should advise patients to exercise** | | **Family/friends should advise patients to exercise** | | **Exercising is easy for patients** | | |
| --- | --- | --- | --- | --- | --- | --- | --- | --- | --- | --- | --- | --- | --- | --- | --- |
|  | **Β (SE)** | **p-value** | **Β (SE)** | **p-value** | **Β (SE)** | **p-value** | **Β (SE)** | **p-value** | **Β (SE)** | **p-value** | **Β (SE)** | **p-value** | **Β (SE)** | **p-value** |  |
| Gender |  |  |  |  |  |  |  |  |  |  |  |  |  |  |  |
| Female | Ref |  | Ref |  | Ref |  | Ref |  | Ref |  | Ref |  | Ref |  |  |
| Male | -0.12 (0.15) | 0.40 | -0.09 (0.15) | 0.55 | -0.14 (0.14) | 0.30 | -0.06 (0.13) | 0.67 | -0.12 (0.14) | 0.40 | -0.12 (0.15) | 0.42 | 0.01 (0.12) | 0.92 |  |
| Age |  |  |  |  |  |  |  |  |  |  |  |  |  |  |  |
| ≤ 39 | Ref |  | Ref |  | Ref |  | Ref |  | Ref |  | Ref |  | Ref |  |  |
| > 39 | -0.14  (0.14) | 0.35 | -0.14  (0.15) | 0.34 | 0.07  (0.14) | 0.60 | 0.12  (0.13) | 0.36 | -0.01  (0.14) | 0.99 | -0.19  (0.15) | 0.20 | 0.26  (0.12) | 0.03 |  |
| Region |  |  |  |  |  |  |  |  |  |  |  |  |  |  |  |
| North | Ref |  | Ref |  | Ref |  | Ref |  | Ref |  | Ref |  | Ref |  |  |
| Central-South | 0.22  (0.19) | 0.26 | 0.14  (0.20) | 0.49 | 0.17  (0.18) | 0.35 | 0.35  (0.18) | 0.04 | 0.21  (0.19) | 0.27 | -0.05  (0.20) | 0.81 | 0.52  (0.16) | 0.002 |  |
| Occupation |  |  |  |  |  |  |  |  |  |  |  |  |  |  |  |
| No | Ref |  | Ref |  | Ref |  | Ref |  | Ref |  | Ref |  | Ref |  |  |
| Yes | 0.01  (0.15) | 0.99 | -0.01  (0.15) | 0.93 | 0.04  (0.14) | 0.77 | -0.09  (0.13) | 0.51 | -0.01  (0.15) | 0.95 | 0.02  (0.15) | 0.91 | -0.06  (0.13) | 0.61 |  |
| Perceived income adequacy |  |  |  |  |  |  |  |  |  |  |  |  |  |  |  |
| Inadequate | Ref |  | Ref |  | Ref |  | Ref |  | Ref |  | Ref |  | Ref |  |  |
| Adequate | 0.28  (0.15) | 0.059 | 0.17  (0.15) | 0.26 | 0.06  (0.14) | 0.64 | 0.05  (0.13) | 0.69 | 0.25  (0.14) | 0.09 | 0.13  (0.15) | 0.37 | 0.15  (0.12) | 0.22 |  |
| Current/prior cancer diagnosis |  |  |  |  |  |  |  |  |  |  |  |  |  |  |  |
| No | Ref |  | Ref |  | Ref |  | Ref |  | Ref |  | Ref |  | Ref |  |  |
| Yes | -0.26  (0.20) | 0.19 | -0.25  (0.20) | 0.20 | -0.03  (0.18) | 0.88 | -0.12  (0.18) | 0.51 | -0.23  (0.19) | 0.24 | 0.01  (0.20) | 0.99 | 0.05  (0.17) | 0.76 |  |
| Leisure Score index |  |  |  |  |  |  |  |  |  |  |  |  |  |  |  |
| ≤ 24 | Ref |  | Ref |  | Ref |  | Ref |  | Ref |  | Ref |  | Ref |  |  |
| > 24 | 0.12  (0.16) | 0.43 | 0.27  (0.17) | 0.08 | 0.26  (0.15) | 0.07 | 0.27  (0.14) | 0.05 | 0.28  (0.15) | 0.07 | 0.31  (0.16) | 0.05 | 0.07  (0.13) | 0.59 |  |

**Table S10.** Multivariable regression of associations of non-healthcare providers' characteristics with their perception regarding exercise in cancer

**Table S11**. Perception of exercise as beneficial according to non-healthcare providers' characteristics

|  | **Disagree, N (%)** | **Neutral, N (%)** | **Agree, N (%)** | **p-value** |
| --- | --- | --- | --- | --- |
| Gender |  |  |  |  |
| Male | 25 (9.9) | 85 (33.6) | 143 (56.5) | 0.024 |
| Female | 60 (13.2) | 111 (24.3) | 285 (62.5) |  |
| Age |  |  |  |  |
| ≤ 39 | 34 (9.6) | 94 (26.6) | 225 (63.7) | 0.089 |
| > 39 | 51 (14.3) | 102 (28.7) | 203 (57.0) |  |
| Region |  |  |  |  |
| North | 69 (11.5) | 178 (29.6) | 354 (58.9) | 0.033 |
| Central-South | 14 (14.0) | 17 (17.0) | 69 (69.0) |  |
| Occupational status |  |  |  |  |
| Employed | 61 (12.2) | 137 (27.3) | 303 (60.5) | 0.947 |
| Not employed | 24 (11.5) | 59 (28.4) | 125 (60.1) |  |
| Financial difficulties |  |  |  |  |
| Yes | 34 (15.1) | 58 (25.8) | 133 (59.1) | 0.204 |
| No | 51 (10.5) | 138 (28.5) | 295 (61.0) |  |
| Current/prior cancer diagnosis |  |  |  |  |
| No | 60 (10.1) | 180 (30.2) | 356 (59.7) | <0.001 |
| Yes | 25 (22.1) | 16 (14.2) | 72 (63.7) |  |
| LSI |  |  |  |  |
| < 24 | 63 (12.5) | 145 (28.7) | 297 (58.8) | 0.366 |
| ≥ 24 | 21 (10.3) | 51 (25.1) | 131 (64.5) |  |

**Table S12**. Perception of exercise as important according to non-healthcare providers' characteristics

|  | **Disagree, N (%)** | **Neutral, N (%)** | **Agree, N (%)** | **p-value** |
| --- | --- | --- | --- | --- |
| Gender |  |  |  |  |
| Male | 24 (9.5) | 82 (32.5) | 146 (57.9) | 0.010 |
| Female | 63 (13.8) | 104 (22.8) | 290 (63.5) |  |
| Age |  |  |  |  |
| ≤ 39 | 38 (10.8) | 84 (23.8) | 231 (65.4) | 0.097 |
| > 39 | 49 (13.8) | 102 (28.7) | 205 (57.6) |  |
| Region |  |  |  |  |
| North | 71 (11.8) | 166 (27.6) | 364 (60.6) | 0.189 |
| Central-South | 14 (14.0) | 19 (19.0) | 67 (67.0) |  |
| Occupational status |  |  |  |  |
| Employed | 63 (12.6) | 131 (26.2) | 306 (61.2) | 0.916 |
| Not employed | 24 (11.5) | 55 (26.3) | 130 (62.2) |  |
| Financial difficulties |  |  |  |  |
| Yes | 35 (15.6) | 50 (22.2) | 140 (62.2) | 0.084 |
| No | 52 (10.7) | 136 (28.1) | 296 (61.2) |  |
| Current/prior cancer diagnosis |  |  |  |  |
| No | 63 (10.6) | 169 (28.4) | 363 (61.0) | <0.001 |
| Yes | 24 (21.1) | 17 (14.9) | 73 (64.0) |  |
| LSI |  |  |  |  |
| < 24 | 64 (12.7) | 145 (28.8) | 295 (58.5) | 0.027 |
| ≥ 24 | 22 (10.8) | 41 (20.1) | 141 (69.1) |  |

**Table S13**. Perception of exercise as safe according to non-healthcare providers' characteristics

|  | **Disagree, N (%)** | **Neutral, N (%)** | **Agree, N (%)** | **p-value** |
| --- | --- | --- | --- | --- |
| Gender |  |  |  |  |
| Male | 27 (10.6) | 138 (54.3) | 89 (35.0) | 0.056 |
| Female | 60 (13.2) | 205 (42.0) | 191 (41.9) |  |
| Age |  |  |  |  |
| ≤ 39 | 38 (10.8) | 184 (52.1) | 131 (37.1) | 0.114 |
| > 39 | 49 (13.7) | 159 (44.5) | 149 (41.7) |  |
| Region |  |  |  |  |
| North | 69 (11.5) | 305 (50.7) | 228 (37.9) | 0.037 |
| Central-South | 16 (16.0) | 37 (37.0) | 47 (47.0) |  |
| Occupational status |  |  |  |  |
| Employed | 62 (12.3) | 241 (47.9) | 200 (39.8) | 0.947 |
| Not employed | 25 (12.1) | 102 (49.3) | 80 (38.6) |  |
| Financial difficulties |  |  |  |  |
| Yes | 35 (15.5) | 99 (43.8) | 92 (40.7) | 0.113 |
| No | 52 (10.7) | 244 (50.4) | 188 (38.8) |  |
| Current/prior cancer diagnosis |  |  |  |  |
| No | 63 (10.6) | 313 (52.5) | 220 (36.9) | <0.001 |
| Yes | 24 (21.1) | 30 (26.3) | 60 (52.6) |  |
| LSI |  |  |  |  |
| < 24 | 65 (12.8) | 256 (50.6) | 185 (36.6) | 0.041 |
| ≥ 24 | 21 (10.3) | 87 (42.9) | 95 (46.8) |  |

**Table S14**. Perception of patients’ capability to perform exercise according to non-healthcare providers' characteristics

|  | **Disagree, N (%)** | **Neutral, N (%)** | **Agree, N (%)** | **p-value** |
| --- | --- | --- | --- | --- |
| Gender |  |  |  |  |
| Male | 36 (14.1) | 153 (60.0) | 66 (25.9) | 0.798 |
| Female | 66 (14.4) | 263 (57.5) | 128 (28.0) |  |
| Age |  |  |  |  |
| ≤ 39 | 49 (13.9) | 214 (60.6) | 90 (25.5) | 0.481 |
| > 39 | 53 (14.8) | 202 (56.3) | 104 (29.0) |  |
| Region |  |  |  |  |
| North | 86 (14.3) | 365 (60.5) | 152 (25.2) | 0.016 |
| Central-South | 14 (13.9) | 48 (47.5) | 39 (38.6) |  |
| Occupational status |  |  |  |  |
| Employed | 75 (14.9) | 292 (58.1) | 136 (27.0) | 0.787 |
| Not employed | 27 (12.9) | 124 (59.3) | 58 (27.8) |  |
| Financial difficulties |  |  |  |  |
| Yes | 43 (18.9) | 114 (50.2) | 70 (30.8) | 0.006 |
| No | 59 (12.2) | 302 (62.3) | 124 (25.6) |  |
| Current/prior cancer diagnosis |  |  |  |  |
| No | 81 (13.5) | 357 (59.6) | 161 (26.9) | 0.249 |
| Yes | 21 (18.6) | 59 (52.2) | 33 (29.2) |  |
| LSI |  |  |  |  |
| < 24 | 74 (14.6) | 304 (60.1) | 128 (25.3) | 0.174 |
| ≥ 24 | 27 (13.2) | 112 (54.6) | 66 (32.2) |  |

**Table S15.** Perception about the role of oncologists in advising patients to perform exercise according to non-healthcare providers' characteristics

|  | **Disagree, N (%)** | **Neutral, N (%)** | **Agree, N (%)** | **p-value** |
| --- | --- | --- | --- | --- |
| Gender |  |  |  |  |
| Male | 27 (10.6) | 102 (40.0) | 126 (49.4) | 0.274 |
| Female | 63 (13.7) | 160 (34.9) | 236 (51.4) |  |
| Age |  |  |  |  |
| ≤ 39 | 34 (9.6) | 141 (39.9) | 178 (50.4) | 0.032 |
| > 39 | 56 (15.5) | 121 (33.5) | 184 (51.0) |  |
| Region |  |  |  |  |
| North | 73 (12.1) | 234 (38.7) | 297 (49.2) | 0.060 |
| Central-South | 15 (14.7) | 27 (26.5) | 60 (58.8) |  |
| Occupational status |  |  |  |  |
| Employed | 60 (11.9) | 191 (37.8) | 254 (50.3) | 0.504 |
| Not employed | 30 (14.4) | 71 (34.0) | 108 (51.7) |  |
| Financial difficulties |  |  |  |  |
| Yes | 38 (16.6) | 78 (34.1) | 113 (49.3) | 0.081 |
| No | 52 (10.7) | 184 (37.9) | 249 (51.3) |  |
| Current/prior cancer diagnosis |  |  |  |  |
| No | 66 (11.0) | 233 (38.8) | 301 (50.2) | 0.002 |
| Yes | 24 (21.1) | 29 (25.4) | 61 (53.5) |  |
| LSI |  |  |  |  |
| < 24 | 67 (13.2) | 198 (38.9) | 244 (47.9) | 0.057 |
| ≥ 24 | 22 (10.8) | 64 (31.4) | 118 (57.8) |  |

**Table S16**. Perception about the role of family/friends in encouraging patients to perform exercise according to non-healthcare providers' characteristics

|  | **Disagree, N (%)** | **Neutral, N (%)** | **Agree, N (%)** | **p-value** |
| --- | --- | --- | --- | --- |
| Gender |  |  |  |  |
| Male | 30 (11.8) | 100 (39.2) | 125 (49.0) | 0.264 |
| Female | 67 (14.6) | 154 (33.6) | 237 (51.7) |  |
| Age |  |  |  |  |
| ≤ 39 | 36 (10.2) | 133 (37.7) | 184 (52.1) | 0.030 |
| > 39 | 61 (16.9) | 121 (33.6) | 178 (49.4) |  |
| Region |  |  |  |  |
| North | 77 (12.8) | 224 (37.1) | 302 (50.1) | 0.120 |
| Central-South | 18 (17.6) | 28 (27.5) | 56 (54.9) |  |
| Occupational status |  |  |  |  |
| Employed | 64 (12.7) | 186 (37.0) | 253 (50.3) | 0.381 |
| Not employed | 33 (15.7) | 68 (32.4) | 109 (51.9) |  |
| Financial difficulties |  |  |  |  |
| Yes | 41 (17.9) | 74 (32.3) | 114 (49.8) | 0.058 |
| No | 56 (11.6) | 180 (37.2) | 248 (51.2) |  |
| Current/prior cancer diagnosis |  |  |  |  |
| No | 74 (12.3) | 230 (38.3) | 296 (49.3) | <0.001 |
| Yes | 23 (20.4) | 24 (21.2) | 66 (58.4) |  |
| LSI |  |  |  |  |
| < 24 | 73 (14.4) | 194 (38.3) | 240 (47.3) | 0.013 |
| ≥ 24 | 23 (11.2) | 60 (29.3) | 122 (59.5) |  |

**Table S17**. Perception of ease for patients to perform exercise according to non-healthcare providers' characteristics

|  | Disagree, N (%) | Neutral, N (%) | Agree, N (%) | p-value |
| --- | --- | --- | --- | --- |
| Gender |  |  |  |  |
| Male | 72 (28.6) | 159 (63.1) | 21 (8.3) | 0.704 |
| Female | 137 (29.9) | 276 (60.3) | 45 (9.8) |  |
| Age |  |  |  |  |
| ≤ 39 | 115 (32.8) | 205 (58.4) | 31 (8.8) | 0.157 |
| > 39 | 94 (26.2) | 230 (64.1) | 35 (9.7) |  |
| Region |  |  |  |  |
| North | 181 (30.2) | 371 (61.8) | 48 (8.0) | 0.017 |
| Central-South | 25 (24.5) | 60 (58.8) | 17 (16.7) |  |
| Occupational status |  |  |  |  |
| Employed | 155 (30.9) | 298 (59.4) | 49 (9.8) | 0.270 |
| Not employed | 54 (26.0) | 137 (65.9) | 17 (8.2) |  |
| Financial difficulties |  |  |  |  |
| Yes | 75 (32.9) | 134 (58.8) | 19 (8.3) | 0.361 |
| No | 134 (27.8) | 301 (62.4) | 47 (9.8) |  |
| Current/prior cancer diagnosis |  |  |  |  |
| No | 178 (29.9) | 362 (60.7) | 56 (9.4) | 0.802 |
| Yes | 31 (27.2) | 73 (64.0) | 10 (8.8) |  |
| LSI |  |  |  |  |
| < 24 | 151 (29.9) | 312 (61.8) | 42 (8.3) | 0.350 |
| ≥ 24 | 57 (27.9) | 123 (60.3) | 24 (11.8) |  |
